# A Connection between Colony Biomass and Death in Caribbean Reef-Building Corals

**DOI:** 10.1371/journal.pone.0029535

**Published:** 2011-12-22

**Authors:** Daniel J. Thornhill, Randi D. Rotjan, Brian D. Todd, Geoff C. Chilcoat, Roberto Iglesias-Prieto, Dustin W. Kemp, Todd C. LaJeunesse, Jennifer McCabe Reynolds, Gregory W. Schmidt, Thomas Shannon, Mark E. Warner, William K. Fitt

**Affiliations:** 1 Department of Field Conservation, Defenders of Wildlife, Washington, District of Columbia, United States of America, and Department of Biology, Bowdoin College, Brunswick, Maine, United States of America; 2 Edgerton Research Laboratory, New England Aquarium, Boston, Massachusetts, United States of America; 3 Department of Wildlife, Fish, and Conservation Biology, University of California, Davis, Davis, California, United States of America; 4 Odum School of Ecology, University of Georgia, Athens, Georgia, United States of America; 5 Instituto de Ciencias del Mar y Limnologia, Universidad Nacional Autonoma de Mexico, Cancun, Mexico; 6 Department of Biology, Pennsylvania State University, University Park, Pennsylvania, United States of America; 8 Department of Ecology and Evolutionary Biology, Tulane University, New Orleans, Louisiana, United States of America; 9 College of Earth, Ocean, and Environment, University of Delaware, Lewes, Delaware, United States of America; King Abdullah University of Science and Technology, Saudi Arabia

## Abstract

Increased sea-surface temperatures linked to warming climate threaten coral reef ecosystems globally. To better understand how corals and their endosymbiotic dinoflagellates (*Symbiodinium* spp.) respond to environmental change, tissue biomass and *Symbiodinium* density of seven coral species were measured on various reefs approximately every four months for up to thirteen years in the Upper Florida Keys, United States (1994–2007), eleven years in the Exuma Cays, Bahamas (1995–2006), and four years in Puerto Morelos, Mexico (2003–2007). For six out of seven coral species, tissue biomass correlated with *Symbiodinium* density. Within a particular coral species, tissue biomasses and *Symbiodinium* densities varied regionally according to the following trends: Mexico≥Florida Keys≥Bahamas. Average tissue biomasses and symbiont cell densities were generally higher in shallow habitats (1–4 m) compared to deeper-dwelling conspecifics (12–15 m). Most colonies that were sampled displayed seasonal fluctuations in biomass and endosymbiont density related to annual temperature variations. During the bleaching episodes of 1998 and 2005, five out of seven species that were exposed to unusually high temperatures exhibited significant decreases in symbiotic algae that, in certain cases, preceded further decreases in tissue biomass. Following bleaching, *Montastraea* spp. colonies with low relative biomass levels died, whereas colonies with higher biomass levels survived. Bleaching- or disease-associated mortality was also observed in *Acropora cervicornis* colonies; compared to *A. palmata*, all *A. cervicornis* colonies experienced low biomass values. Such patterns suggest that *Montastraea* spp. and possibly other coral species with relatively low biomass experience increased susceptibility to death following bleaching or other stressors than do conspecifics with higher tissue biomass levels.

## Introduction

Coral reefs are highly diverse, productive, and economically-important ecosystems found in shallow marine environments throughout the world's tropical and sub-tropical oceans. Currently, populations of reef-building corals are in global decline due to increased sea-surface temperatures associated with climate change [1]. The worldwide degradation of coral reefs has been further exacerbated at local and regional scales by an array of anthropogenic stressors, such as nutrient inputs, overfishing, destructive fishing practices, exotic species introductions, increased sedimentation and pollution [2], [3]. As a result of this ecological crisis, there is an urgent need to understand how acute and chronic stressors affect the physiological integrity of reef-building corals.

Perhaps the greatest of the problems facing reefs today is the warming of sea-surface temperatures that, in conjunction with intense solar radiation, causes widespread coral bleaching and mortality [1]. Bleaching is defined as the loss of endosymbiotic dinoflagellates in the genus *Symbiodinium* (a genetically diverse group that is commonly known as zooxanthellae) from the coral's tissue, but it may also include loss of pigments by the endosymbiont and/or the host [4]. Coral bleaching is often associated with El Niño Southern Oscillation (ENSO) events and climate change [1]. Because reef-building corals receive energy from their *Symbiodinium* communities [5], severe bleaching often results in coral colony death through starvation or disease [1], [6], [7]. For example, an intense ENSO event in 1997–1998 led to loss of approximately 16% of coral reefs globally, including corals in the Caribbean Sea [8].

Surveys of mass bleaching events have documented differential mortality among coral colonies and species [6], [9]. In some cases, neighboring colonies of the same coral species were differentially affected by bleaching [10]. The factors underlying differential mortality of corals under seemingly identical conditions of depth and reef position remain poorly understood. It has been proposed that this response is due to a combination of genotypic and physiological variation among coral colonies [11] and/or their endosymbionts [12], [13]. One additional factor affecting post-bleaching mortality is a colony's level of energy reserves, which is correlated with tissue biomass or ash-free dry weight (AFDW).

Coral colony biomass is the product of a complex array of factors. Reef-building corals obtain energy from polytrophic sources, including photosynthetic endosymbionts, heterotrophic feeding, and absorption of dissolved organic matter (DOM), and this energy is allocated to metabolism, reproduction, skeletal growth (i.e., calcification), mucus production, tissue repair and defense, and production of new biomass (e.g., tissue growth and energy reserves) [14]–[16]. A colony's biomass level thus depends upon the equilibrium between various energetic sources vs. expenditures and is subject to dynamic change over time. Such change can be observed in the fluctuations of coral tissue biomass and *Symbiodinium* population density in response to seasonal variation in temperature and light, with both parameters reaching annual nadir during the hottest times of the year [17], [18]. Tissue biomass and *Symbiodinium* density are further depleted during mass bleaching events [18], which jeopardize colony survival due to corals' reliance on energy reserves for sustenance during stress. If biomass reserves and energy inputs from autotrophic symbionts, heterotrophic feeding, and absorption are insufficient, then a colony may perish from starvation or due to secondary stressors (e.g., disease).

**Figure 1:**
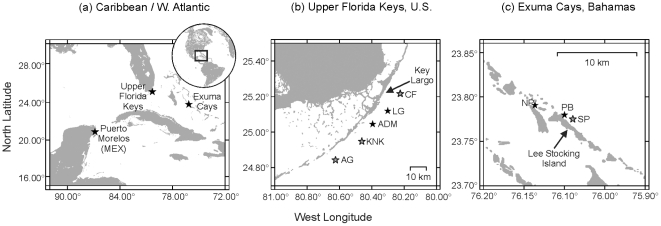
Regional and local maps of collection sites for this study. Coral collection depth is indicated in panels b and c by star shading; shallow reefs (1–4 m) are indicted by a black star whereas deep reefs (12–15 m) are denoted by a gray star. Reef abbreviations are as follows: Upper Florida Keys, U.S.: CF = Carysfort Reef, LG = Little Grecian Reef, ADM = Admiral Patch Reef, KNK = Conch Reef, AG = Alligator Reef; Exuma Cays, Bahamas: NP = North Norman's Patch Reef, PB = Palmata Beach Reef, SP = South Perry Reef; Puerto Morelos, Mexico: MEX = UNAM. Figure adapted from Thornhill et al. [20].

Based on the above, colony biomass may be an indicator of coral health and resilience to stress. If this hypothesis is correct, biomass differences among colonies and species across a coral reef could explain inter-colony differences in bleaching mortality. Here, we examine this hypothesis and add additional perspectives on coral physiology and the bleaching phenomenon from long-term analysis of tissue biomass and the density of endosymbionts in seven species of reef-building corals. Corals were collected from nine different reefs spanning three geographic regions from a time span of 3–13 years (Fig. 1, Table 1). Our goals were to 1) examine the relationship between tissue biomass and *Symbiodinium* density, 2) determine how these two parameters vary spatially (i.e., by region, habitat, and depth) and temporally (i.e., season), 3) assess changes in these two parameters following two bleaching events (1998 and 2005), and 4) determine the relationship between coral tissue biomass and colony mortality.

**Table 1:** Species of corals sampled at each reef in this study.

Region	Reef	Latitude	Longitude	Depth	Coral Species	Duration Sampled
Exuma Cays, Bahamas	Norman's Pond (NP)	23.79°N	76.14°W	0–4 m	*M. annularis*	1995–2006
					*M. faveolata*	1995–2006
					*A. cervicornis*	2001–2006
					*P. astreoides*	2002–2003
					*S. siderea*	2002–2005
	Palmata Beach (PB)	23.78°N	76.10°W	0–4 m	*A. cervicornis*	1996–2005
					*A. palmata*	1995–2005
	South Perry (SP)	23.77°N	76.09°W	12–15 m	*M. annularis*	1995–2006
					*M. faveolata*	1995–2006
					*M. franski*	1997–2006
					*A. cervicornis*	1996–2006
Upper Florida Keys, USA	Little Grecian Reef (LG)	25.12°N	80.30°W	0–4 m	*M. annularis*	1999–2007
					*M. faveolata*	1999–2007
					*A. cervicornis*	1996–2007
					*A. palmata*	1996–2007
					*S. siderea*	2002–2007
	Admiral Reef (ADM)	25.05°N	80.39°W	0–4 m	*M. annularis*	1995–2007
					*M. faveolata*	1994–2007
					*A. cervicornis*	1996–2007
					*P. astreoides*	2002–2007
					*S. siderea*	2002–2007
	Carysfort Reef (CF)	25.22°N	80.23°W	12–15 m	*M. annularis*	1995–1999
					*M. faveolata*	1996–1999
					*M. franski*	1997–1999
					*A. cervicornis*	1996–1998
	Conch Reef (KNK)	24.94°N	80.46°W	12–15 m	*M. annularis*	1999–2001
					*M. faveolata*	2000–2002
					*M. franksi*	1999–2002
	Alligator (AG)	24.84°N	80.62°W	12–15 m	*M. annularis*	2003–2007
					*M. faveolata*	2003–2007
					*M. franksi*	1997–2007
					*A. cervicornis*	2003–2005
Puerto Morelos, Mexico	UNAM (MEX)	20.87°N	86.85°W	0–4 m	*M. annularis*	2003–2007
					*M. faveolata*	2003–2007
					*P. astreoides*	2003–2007

## Results

**Figure 2:**
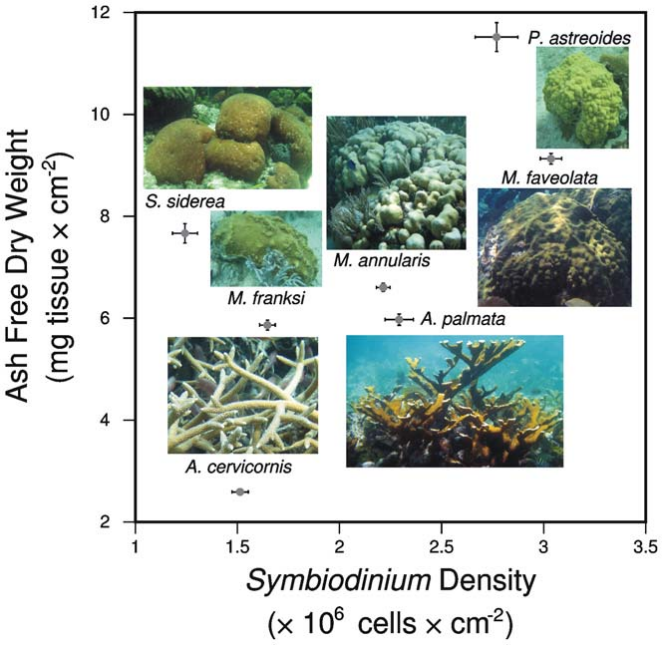
Species-specific values for tissue biomass versus *Symbiodinium* density from seven species of scleractinian corals. Biomass was measured by ash-free dry weight in mg tissue×cm^−2^ whereas *Symbiodinium* density was quantified as cells×10^6^×cm^−2^. Data presented as mean values ± standard error. A photograph of each coral species is provided for reference. Photographs are not to scale. Photo credits: D.J. Thornhill, G.C. Chilcoat, and D.W. Kemp.

Each coral species exhibited a species-specific mean tissue biomass and symbiont density value (Fig. 2). *Porites astreoides* had the highest mean coral tissue biomass (11.52±0.28 mg tissue×cm^−2^; data presented as mean ± SE throughout this manuscript), whereas the lowest biomass was found in *Acorpora cervicornis* (2.59±0.05 mg tissue×cm^−2^). The highest and lowest *Symbiodinium* densities occurred in *Montastraea faveolata* (3.04±0.05 cells×10^6^×cm^−2^) and *Siderastrea siderea* (1.24±0.06 cells×10^6^×cm^−2^), respectively. Data for the remaining coral species fell between these extremes of ash free dry weight and symbiont density (Fig. 2).

**Figure 3:**
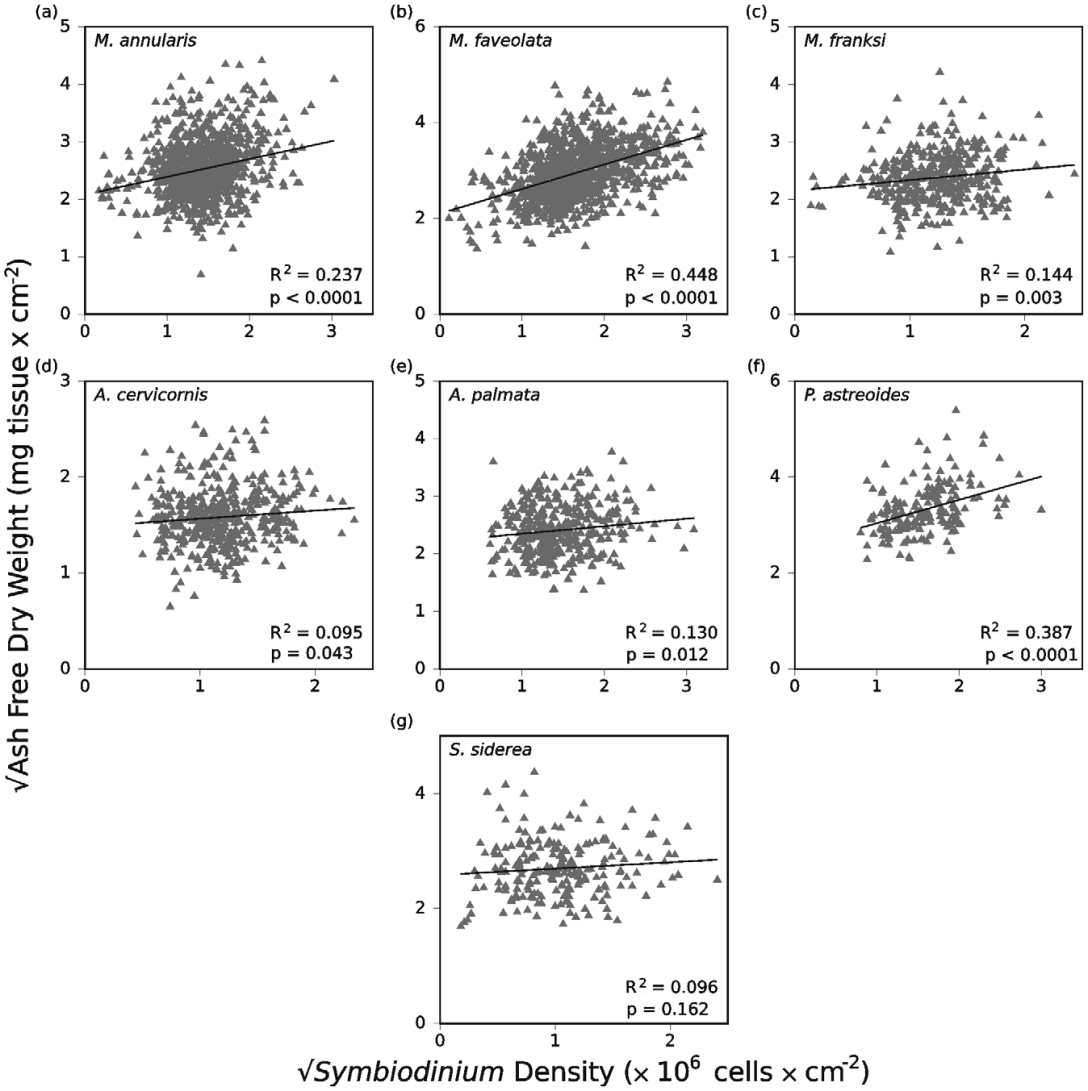
Scatter plots depicting the relationship between tissue biomass versus *Symbiodinium* density for seven reef-building corals. Data have been square root transformed in order to meet normality assumptions and a trend line has been fitted to the transformed dataset for each species. Coral species identity (upper left), degree of correlation (R^2^ value, lower right), and p value (lower right) are provided for each species.

For six of the seven reef-building coral species, tissue biomass and *Symbiodinium* density were positively and significantly correlated with one another (Fig. 3). The exception was *S. siderea*, where biomass and symbiont density were not correlated (*r* = 0.096, p = 0.162, Fig. 3g). Among the remaining six species, the degree of correlation between the two parameters varied considerably (*r* = 0.095–0.448). The correlation between tissue biomass and *Symbiodinium* density was strongest for *M. faveolata* (*r* = 0.448, p<0.0001) and *P. astreoides* (*r* = 0.387, p<0.0001), whereas the relationship was considerably weaker in *A. cervicornis* (*r* = 0.095, p = 0.043) relative to the other five coral species (Fig. 3). The remaining three species had correlations of intermediate strength, including *A. palmata* (*r* = 0.130, p = 0.012), *M. annularis* (*r* = 0.237, p<0.0001), and *M. franksi* (*r* = 0.144, p = 0.003).

In all seven coral species, the correlation coefficients suggested that much of the relationship between tissue biomass and *Symbiodinium* density was left unexplained. In the results that follow we examine other factors that affect this variation by examining how tissue biomass and endosymbiont density vary spatially among regions, depths and reefs, temporally across seasons, following two mass bleaching events, and in relation to coral colony mortality.

### Regional and depth patterns

**Figure 4:**
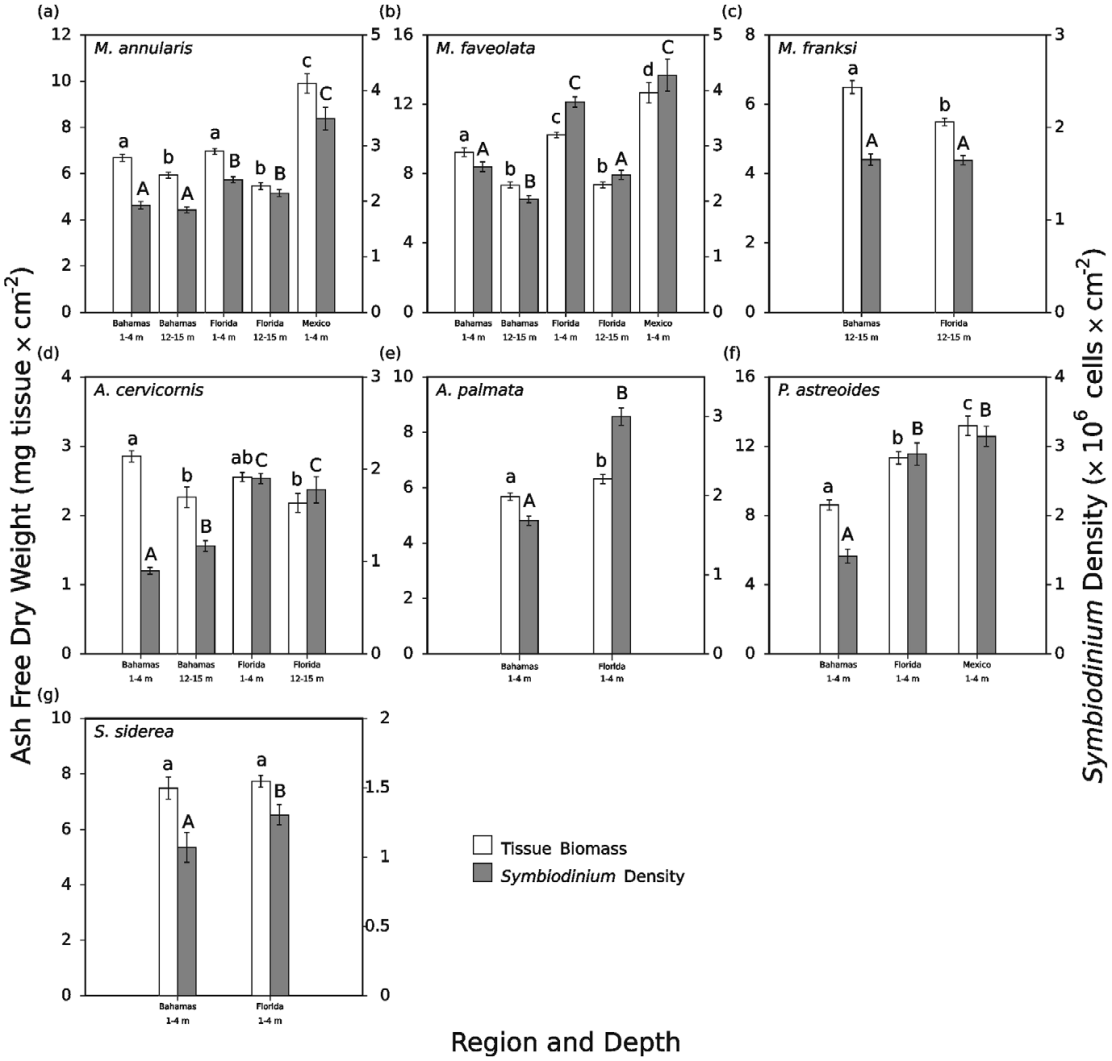
Coral tissue biomass and *Symbiodinium* density by region and depth within regions. Coral tissue biomass (left axis and white bars) and *Symbiodinium* density (right axis and shaded bars) data presented as mean values ± standard error. Statistically distinguishable versus indistinguishable patterns (at α = 0.05) are indicated letters above the histograms for each species (using lower case letters for tissue biomass and upper case letters for *Symbiodinium* densities). Statistical analyses conducted by either two-factor ANOVAs (for *M. annularis*, *M. faveolata*, *A. cervicornis*, and *P. astreoides*) or T-tests (for *M. franksi, A. palmata,* and *S. siderea*). Coral species identity is indicated (upper left) for each sub-panel.

Patterns of coral tissue biomass and symbiont density varied geographically among the Bahamas, the Florida Keys (U.S.), and Mexico for many of the coral species examined (Fig. 4). For example, at shallow depths (1–4 m), corals from the Bahamas often had lower mean tissue biomass and/or *Symbiodinium* density values than conspecifics from other regions (Fig. 4). This pattern occurred in the endosymbiont cell densities for *M. annularis* (p values<0.0001), *A. cervicornis* (p<0.0001), and *S. siderea* (p = 0.039, two-sample t-test), as well as in both parameters for *M. faveolata* (p values<0.0001), *A. palmata* (p values≤0.004, two-sample t-test), and *P. astreoides* (p values<0.0001) (Fig. 4, unless otherwise noted, regional and depth comparisons and hypotheses testing conducted via two-factor ANOVA and Tukey's honestly-significant-difference tests). By contrast, values for the tissue biomass and *Symbiodinium* density of shallow water corals in the Florida Keys were either intermediate to those of the Bahamas and Mexico (e.g., *M. faveolata* tissue biomass p values<0.001; *M. annularis* symbiont density p values<0.0001; *P. astreoides* tissue biomass, Florida vs. Mexico p = 0.004, Florida vs. the Bahamas p<0.001), statistically indistinguishable from those of the Bahamas (e.g., *M. annularis,* tissue biomass p = 0.827; *S. siderea* tissue biomass two sample t-test p = 0.465), or statistically indistinguishable from those of Mexico (e.g., *M. faveolata Symbiodinium* density p = 0.554; *P. astreoides Symbiodinium* density p = 0.258) (Fig. 4). Finally, shallow *M. annularis*, *M. faveolata*, and *P. astreoides* from Mexico had the highest mean tissue biomasses and/or symbiont densities relative to conspecifics from other regions (Fig. 4, p values<0.0001 except *M. faveolata* Florida vs. Mexico symbiont density where p = 0.554 and *P. astreoides* Florida vs. Mexico where tissue biomass p = 0.004 and symbiont density p = 0.258). Thus, the regional tissue biomass and *Symbiodinium* density patterns at shallow depths (1–4 m) can be summarized as follows: Mexico≥Florida≥Bahamas (Fig. 4).

At depths of 12–15 m, comparisons between regions were only possible in the Bahamas and Florida Keys for four of the species (i.e., *M. annularis*, *M. faveolata*, *M. franksi*, and *A. cervicornis*). At deep reefs, *M. annularis*, *M. faveolata*, and *A. cervicornis* exhibited mean tissue biomasses that did not differ significantly (p>0.05) between regions (Fig. 4). *Montastraea franksi* was an exception to this pattern, having higher mean tissue biomass in the Bahamas relative to the Florida Keys (Fig. 4c, two sample t-test p<0.0001). *Symbiodinium* densities either did not differ significantly between regions (i.e., *M. franksi* two sample T-test p = 0.923) or were higher in Florida than in the Bahamas (i.e., for *M. annularis* p = 0.002, *M. faveolata* p = 0.003, and *A. cervicornis* p = 0.003; Fig. 4). Therefore, at greater depth, the typical regional tissue biomass patterns were Florida = Bahamas, with the exception of *M. franksi.* The generalized regional *Symbiodinium* density patterns were Florida≥Bahamas.

As noted above, within a region and coral species, patterns of coral tissue biomass and symbiont density frequently varied by depth (Fig. 4). As with geographic comparisons, depth-based comparisons were limited by the natural occurrence of the target species and sampling design of this study. Therefore, inter-depth comparisons were only possible for *M. annularis*, *M. faveolata*, and *A. cervicornis* within the Bahamas and Florida Keys. For the *Montastraea* species, mean tissue biomass was significantly lower in deep-dwelling corals relative to shallow conspecifics in both the Bahamas and Florida (Fig. 4, p values<0.0001). In contrast, shallow and deep *A. cervicornis* from Florida (p = 0.201), but not the Bahamas (p = 0.001), had mean tissue biomasses that did not differ significantly (Fig. 4).

Within a given region, *Symbiodinium* densities either did not differ significantly (i.e., for *M. annularis* [Bahamas p = 0.908, Florida p = 0.145] and Florida *A. cervicornis* [p = 0.696]) or were lower at depth when compared to their shallow water counterparts (i.e., for *M. faveolata* p values<0.0001). However, *A. cervicornis* in the Bahamas was exceptional in that deep-water *A. cervicornis* exhibited significantly higher (p = 0.007) symbiont densities than did shallow conspecifics (but note the low sample size at deep reefs). In summary, mean AFDW values and symbiont densities could be generalized as shallow (1–4 m)≥deep (12–15 m), with the exception of *A. cervicornis* endosymbiont densities in the Bahamas.

### Seasonal patterns

**Figure 5:**
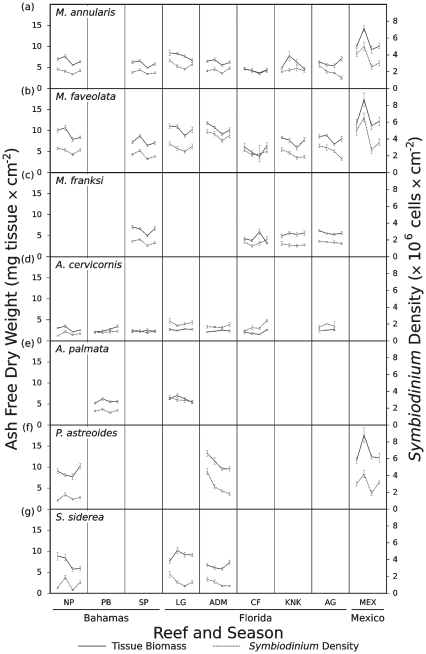
Seasonal variation in coral biomass and *Symbiodinium* density for seven coral species from nine reefs. Coral tissue biomass (left axis and solid lines) and *Symbiodinium* density (right axis and dashed lines) data presented as mean values for a given season ± standard error. Within each line plot, four sample dates are provided, one each for winter (January to March), spring (April to June), summer (July to September) and fall (October to December). Seasonal means are presented chronologically beginning with winter and ending with fall. A blank sub-panel indicates that a particular coral species did not occur at that specific location. Reef abbreviations provided in Fig. 1. Statistical analyses of these data are given in Table S1.

The sampling design of this study also included examination of seasonal fluctuations in tissue biomass and *Symbiodinium* density (i.e., from winter to spring to summer to fall). Mean seasonal tissue biomass and symbiont density values are presented for each coral species and reef in Fig. 5 and a summary of the statistical analyses performed on these data is provided in Table S1. Both coral tissue biomass and endosymbiont cell density varied seasonally, with the highest values often occurring in the winter or spring and lowest values frequently occurring during the summer or fall (Fig. 5). Notably, significant variation was detected among reefs, among seasons, and among the interaction of reef and season for both physiological parameters in four of the seven coral species sampled, including *M. annularis*, *M. faveolata*, *P. astreoides* (endosymbiont density only), and *S. siderea* (Table S1). Variation among reefs reflected the differences between regions and depths explored above (Fig. 4), as well as differences between the specific sites sampled (Fig. 5). Variation among seasons was typically due to high tissue biomass and symbiont density in the winter/spring and low values in the summer/fall.

Four coral species deviated from the seasonal patterns described above. Deep-dwelling *M. franksi* exhibited significant tissue biomass variation among reefs, but not between seasons or in the interaction between reefs and seasons (Table S1, Fig. 5c). The lack of significant seasonality stems from a combination of no seasonal change at certain sites and asynchronous seasonality among other sampled reefs. For *M. franski Symbiodinium* densities, significant variation occurred seasonally as well as in the interaction between reef and season, but not among reefs alone (Table S1, Fig. 5c). The second exception to the seasonality described above was in both the tissue biomass and *Symbiodinium* densities of *A. cervicornis.* Here, statistical analyses revealed significant variation between reefs, but no effect of season or of the interaction between season and reef (Table S1). As illustrated in Fig. 5d, the tissue biomass and symbiont densities of *A. cervicornis* generally remained constant and low relative to other species throughout the year. For *A. palmata*, significant variation was detected between reefs for both parameters and significant seasonality was detected in tissue biomass (Table S1). However, there was no significant seasonality in *A. palmata Symbiodinium* density or in the interaction between reef and season for either parameter (Table S1). This may have resulted from the asynchronous seasonality between reefs for *A. palmata Symbiodinium* density and the relatively low overall fluctuations in this coral (Fig 5e). Finally, the tissue biomass of *P. astreoides* varied significantly among reefs and in the interaction between reef and season, but not significantly by season alone (Table S1). The lack of significant seasonal variation in *P. astreoides* biomass may result from seasonal highs occurring at different times of the year for each of the reefs sampled (i.e., asynchronous seasonality, Fig. 5f). Beyond these three exceptions, tissue biomass and *Symbiodinium* density fluctuated significantly across seasons for the sampled coral species (Table S1).

### The 1998 and 2005 coral bleaching episodes

Two major bleaching episodes occurred in the Caribbean Sea and western Atlantic Ocean during the study. In 1998, tropical coral reefs across the world, including reefs of the Florida Keys, Bahamas, and Mexico, experienced a devastating mass bleaching event associated with elevated sea-surface temperatures [1], [8]. Although circumstances prevented the sampling of some Florida reefs during this bleaching event and sampling had not yet begun in Mexico, data were collected for many of the colonies and species under investigation in the Bahamas and for *Montastraea* spp. at ADM reef in Florida. A second bleaching event occurred in 2005 in the Bahamas and Mexico, but not on the reefs we sampled in Florida [19], [20].

**Figure 6:**
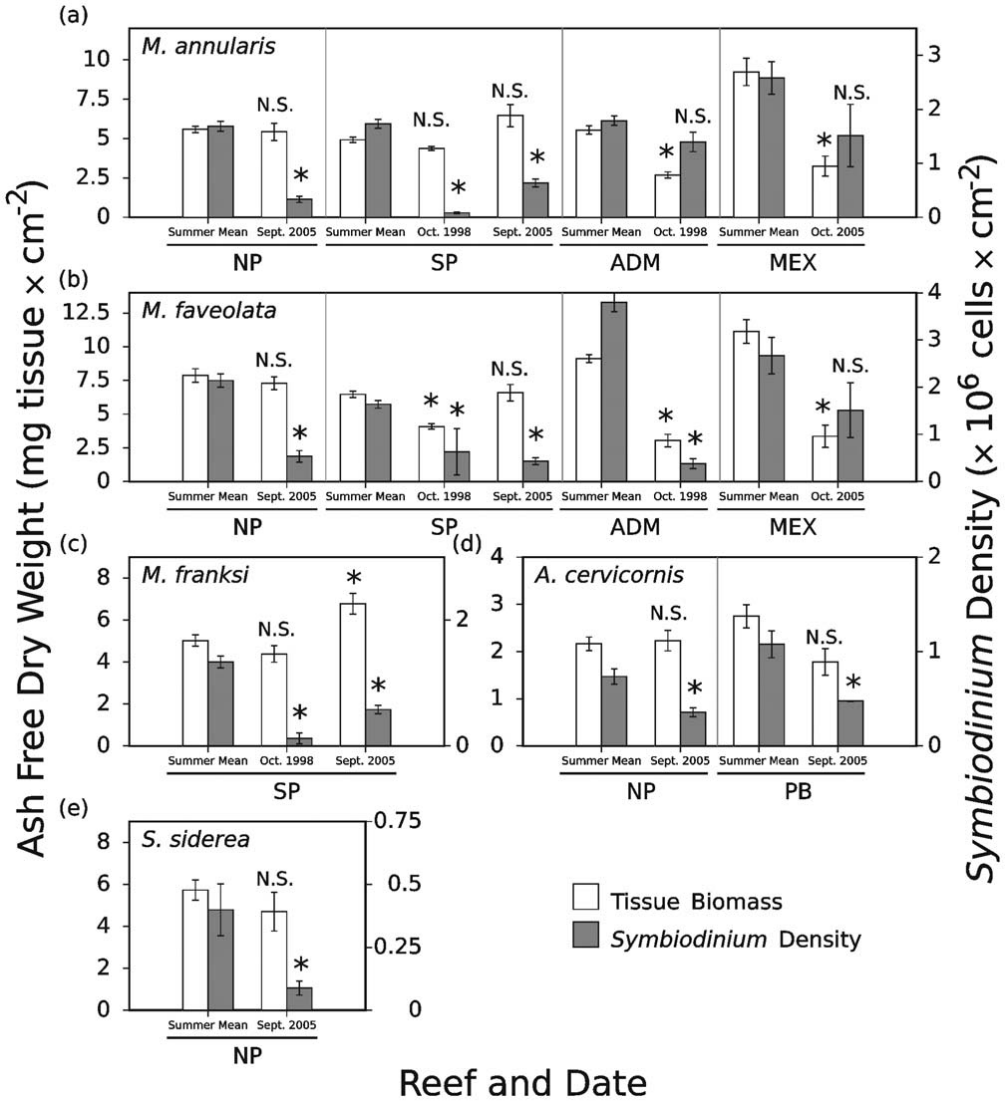
Changes in *Symbiodinium* density and tissue biomass coinciding with bleaching events for five coral species. Data are presented as mean values of tissue biomass (left axis and white bars) and *Symbiodinium* density (right axis and shaded bars) ± standard error. Significant deviation from the mean summer tissue biomass and *Symbiodinium* density, as evaluated using T-tests, indicated above the histograms for each species (N.S. = no significant difference, * = significant deviation from the summer mean at α = 0.05). Reef abbreviations provided in Fig. 1. Note that bleaching impacts are not provided for certain coral species due to either a lack of sampling during the bleaching events (e.g., certain corals from the Florida Keys during the 1998 bleaching) or due to a lack of visible and statistically significant bleaching for that species and location (e.g., *A. palmata* from all regions in 2005).

Changes in *Symbiodinium* density and tissue biomass in relation to the 1998 and 2005 bleaching events are presented in Fig. 6. For *M. annularis*, *M. faveolata*, *M. franksi*, *A. cervicornis*, and *S. siderea* from the Bahamas, bleaching was characterized by statistically significant reductions in *Symbiodinium* density relative to the normal mean summer minima for a given coral species and location (p<0.001 for all comparisons with the exception of *S. siderea* where p = 0.004; Fig. 6). The remaining two species, *A. palmata* and *P. astreoides*, did not experience reductions in their endosymbiont densities. In Florida, *M. faveolata* at ADM reef experienced significant reductions in *Symbiodinium* density (p<0.0001) during the bleaching event. However, changes in *Symbiodinium* density were not significant in *M. annularis* at ADM reef (p = 0.164). No statistically significant change in *Symbiodinium* density was detected for *M. annularis* (p = 0.124) or *M. faveolata* (p = 0.184; Fig. 6a–b) in Mexico in 2005, likely because the site was sampled approximately two weeks into recovery from bleaching, following a cold front.

In contrast to symbiont densities, tissue biomass values were less often correlated with bleaching in the immediate aftermath of the 1998 and 2005 events (Fig 6). Nevertheless this, visible bleaching was linked in a few cases to reduced tissue biomass, including *M. annularis* from ADM reef (p<0.0001) in 1998, SP reef in 2005 (p = 0.04), and Mexico in 2005 (p<0.0001), as well as in *M. faveolata* from ADM reef in 1998 (p<0.0001), SP reef in 1998 (p<0.0001), and Mexico in 2005 (p = 0.002) (Fig. 6).

### Coral colony mortality and tissue biomass

**Figure 7:**
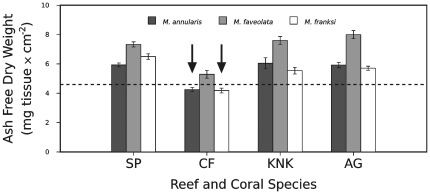
Tissue biomass of *Montastraea* spp. at 12–15 m depth in relation to colony mortality. A black arrow indicates that all coral colonies of that species and location died during the course of this study. Data presented as mean values of tissue biomass ± standard error. Coral species identity indicated by bar color (see legend). Reef abbreviations provided in Fig. 1. Dashed line indicates the estimated threshold for mortality susceptibility (see text).

The majority of coral colonies survived throughout the study period, including through the 1998 and 2005 bleaching events. However, colony mortality was observed in *M. annularis* and *M. franksi* from CF reef in the Florida Keys in the months following the 1998 bleaching (black arrows, Fig. 7). *Montastraea annularis* and *M. franksi* colonies that died at CF reef had consistently lower mean tissue biomass values relative to *M. faveolata* from CF reef, and relative to conspecific and congeneric corals from other deep (12–15 m) reefs (Figs. 5, 7). The low tissue biomass of *M. annularis* and *M. franksi* at CF reef predated the 1998 bleaching event and no sudden dip in biomass levels was observed after the 1998 bleaching. Instead, the *Montastraea* spp. corals with a low tissue biomass simply did not recover from bleaching and these colonies died within 4 months of the event.

**Figure 8:**
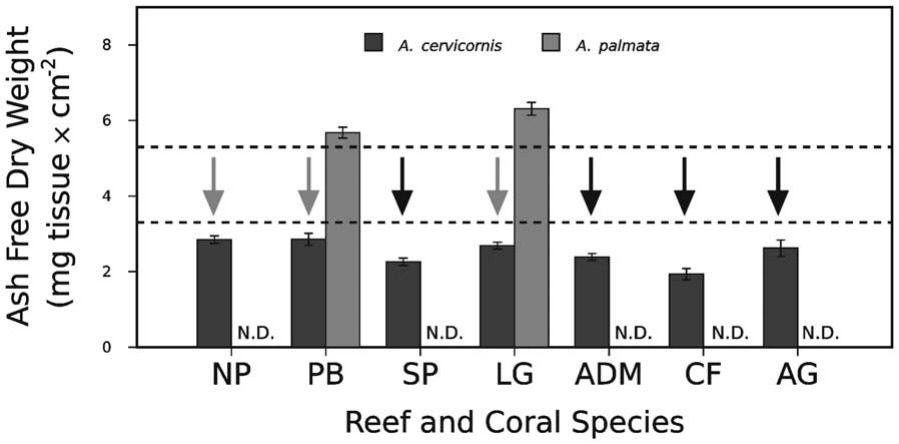
Tissue biomass of *Acropora* spp. in relation to colony mortality. A black arrow indicates that all coral colonies of that species and location died during the course of this study. Gray arrows indicate substantial reductions in live coral tissue for all coral colonies from that location. Data presented as mean values of tissue biomass ± standard error. Coral species is indicated by bar color (see legend). The designation ‘N.D.’ (i.e., no data) denotes the lack of a particular coral species at a specific location. Reef abbreviations provided in Fig. 1. Dashed lines demarcate the statistically significant range of thresholds for mortality susceptibility (see text).

Coral colony death also occurred in 100% of the *A. cervicornis* colonies sampled from SP, ADM, CF, and AG reefs (black arrows, Fig. 8). At NP, PB, and LG reefs, *A. cervicornis* colonies experienced reductions in the surface area of living tissue and death of numerous colonies by the conclusion of sampling in 2006–2007 (gray arrows, Fig. 8). Mortality in *A. cervicornis* was associated with the 1998-bleaching event at CF reef. Because of the high incidence of mortality and tissue decline, conspecific tissue biomass comparisons between healthy and dying corals were not possible for *A. cervicornis*. However, it is noteworthy that the mean tissue biomass values for *A. cervicornis* were substantially lower than the mean biomasses of its sister taxon, *A. palmata*, regardless of the reef, depth, or region sampled (Fig. 8). None of the sampled *A. palmata* died over the duration of this study.

The correlations between colony mortality and low tissue biomass in *M. annularis*, *M. franksi*, and potentially *A. cervicornis*, led to the following hypothesis: low tissue biomass renders coral colonies susceptible to death if a stressful event, such as bleaching or disease, should occur. If correct, the threshold for mortality susceptibility is species or genera specific. Specifically, *M. annularis* and *M. franksi* at CF reef that died had mean tissue biomasses of 4.25±0.14 and 4.19±0.16 mg tissue×cm^−2^, respectively (Fig. 7). By contrast, the mean tissue biomass for *A. cervicornis* ranged between 1.94±0.15 mg tissue×cm^−2^ at CF reef to 2.85±0.10 mg tissue×cm^−2^ at NP reef (Fig. 8).

To mathematically represent this minimum tissue biomass hypothesis, mortality thresholds for *Montastraea* were calculated (in increments of 0.1 mg tissue×cm^−2^) between the mean tissue biomass values for *M. faveolata* at CF reef (which survived with a mean value of 5.30±0.26 mg tissue×cm^−2^) and those of *M. annularis* and *M. franksi* from CF reef (which perished). Consistent with our minimum tissue biomass hypothesis, the value of 4.6 mg tissue×cm^−2^ was found to be the optimal threshold for *Montastraea* spp. (Fig. 7; Text S1). Threshold analysis was also conducted for *Acropora* spp., and a range was determined between the values of 2.9 and 5.6 mg tissue×cm^−2^. This range covered the maximum tissue biomass observed among dying *A. cervicornis* (at NP and PB) and the minimum in surviving *A. palmata* (at PB) respectively. For *Acropora* spp., values ranging between 3.3 and 5.3 mg tissue×cm^−2^ represented acceptable thresholds for mortality; the optimal threshold was 4.0 (Fig. 8; Text S1).

## Discussion

Examination of coral tissue biomass and *Symbiodinium* density from seven species of reef-building corals from the Caribbean Sea and western Atlantic Ocean revealed a number of statistically robust patterns. Specifically, coral species displayed a species-specific mean biomass and endosymbiont density, as well as a positive and significant correlation between these two parameters (with the exception of *S. siderea*). Tissue biomass and *Symbiodinium* density often varied over both space and time, with a tendency towards the following generalized patterns: Mexico≥Florida≥Bahamas, shallow≥deep, and winter/spring>summer/fall. The 1998 and 2005 bleaching events were characterized by reduced *Symbiodinium* densities, but not necessarily host tissue biomass, for many coral species. Finally, colonies with relatively low tissue biomass often succumbed to death when exposed to thermal stress and/or disease. In the following discussion, we consider how tissue biomass and symbiont cell density relate to geography, depth, seasonal variation, bleaching history, and differential mortality.

### Tissue biomass and Symbiodinium density are positively correlated

Six out of seven reef-building coral species examined here exhibited a correlation between *Symbiodinium* density and tissue biomass. One possible explanation for such correlations is that endosymbionts are an important component of overall tissue biomass. This possibility has not been explored in the published scientific literature and would make an important topic for future studies. However, data made available to us by S.C. Kempf provide insight into the contributions of *Symbiodinium* to tissue biomass. Kempf separated host and endosymbiont tissues in five symbiotic cnidarians from the Florida Keys and measured the ash free dry weight of isolated *Symbiodinium* cells (S.C. Kempf unpublished data). For *M. annularis*, that analysis resulted in tissue biomass values of 131±4 pg×*Symbiodinium* cell^−1^ (S.C. Kempf unpublished data; n = 10 symbiont cell sub-samples; value presented as mean ± SD). Based on the cell densities recovered in our study and the cell biomass values measured by S.C. Kempf, we estimate that *Symbiodinium* cell biomass accounts for less than 5% of the *M. annularis* AFDW value. Assuming that a similar biomass of *Symbiodinium* cells occurs in other reef-building coral species, it appears that symbionts did not appreciably bias the measurement of host AFDW. This conclusion is further supported in many coral species by both the weak relationships between symbiont density and biomass and the immediate decreases in *Symbiodinium* densities seen during or just after bleaching events with a subsequent loss of tissue biomass delayed until the next sampling point. Nevertheless, it is possible that the relative contributions of *Symbiodinium* vary among different host and/or endosymbiont species and/or in space and time. Additional study is clearly needed to better document this relationship. Until such data are available, biological inferences about the correlations between symbiont density and overall biomass should be considered with caution.

Resident populations of photosynthetic dinoflagellates support the metabolic needs of most reef-building corals [5], [21], [22]. Photosynthetic material is translocated in excess to host corals, especially during the cooler seasons [18], [23]. Host tissue biomass in corals such as *M. faveolata* and *P. astreoides* were greatest when endosymbiont densities were at their highest, despite the fact that *Symbiodinium* themselves comprise only a marginal portion of measured AFDW (see above). Thus, nutritional (or energetic) mutualisms between corals and symbiotic dinoflagellates likely drive the positive correlations between *Symbiodinium* densities and host tissue biomasses for most (i.e., six out of seven) species examined here. Presumably, coral species with a weak or uncorrelated biomass to *Symbiodinium* density relationship, such as *A. cervicornis* and *S. siderea*, either obtain a larger proportion of their energetic needs through alternative sources (i.e., heterotrophic feeding and/or absorption), harbor *Symbiodinium* populations whose net carbon translocation is uncoupled from cell density, allocate more of their energy to other biological requirements (e.g., calcification, mucus production, reproduction, etc.), or some combination of these scenarios.

Additional factors probably contribute to the variation in AFDW and densities of endosymbiotic dinoflagellates. Individuals, populations, and species of coral living under various habitats or associating with different symbiont genotypes many differentially rely on photosynthesis, heterotrophy, and DOM absorption depending on their circumstances [24], [25]. In Hawaii, for instance, the coral *Montipora capitata* compensates for stress-related reductions in *Symbiodinium* density through increased feeding, whereas *Porites compressa* and *P. lobata* do not [26]. Physiological and morphological variation among *Symbiodinium* may partially explain the differences in AFDW and symbiont cell density among host species. Certain *Symbiodinium* lineages presumably transfer less photosynthate to their hosts than do others [27], which may translate into different degrees of correlation between coral biomass and symbiont density among different coral species. Previous work confirms the presence of considerable genetic diversity of *Symbiodinium* among the colonies and species examined here [19], [20], [28]. More precise information about the physiological contributions of different *Symbiodinium* lineages is required to determine the degree of mutualism in various host-symbiont combinations.

### Tissue biomass and Symbiodinium density vary regionally

The regional differences in tissue biomass and *Symbiodinium* density encountered here likely relate to latitudinal changes in light and temperature, as well as bleaching history. Tissue biomass and *Symbiodinium* densities can diminish as irradiance decreases; for instance, across a depth range [18] or with increased shading in underwater caves [29]. It is therefore possible that such differences might be evident in colonies living in high versus low latitudes. Puerto Morelos, Mexico is located at lower latitude than the other regions sampled resulting in more direct photosynthetically active radiation exposure and less seasonal variation. This increased photosynthetically active radiation and seasonal stability may explain the higher tissue biomass in *M. annularis*, *M. faveolata* and *P. astreoides* from Mexico, relative to other regions.

If latitudinal differences in PAR and temperature are responsible for the geographic differences, it is surprising that tissue biomass and endosymbiont cell densities were occasionally lower in the Exuma Cays, Bahamas relative to conspecifics in the Upper Florida Keys, U.S. Although the regions are located at similar latitudes, the Exuma Cays are farther south than the Florida Keys and receive slightly greater light exposure and lower seasonal variation in environmental conditions [30]. Additionally, reefs of the Upper Florida Keys are proximate to large human populations, presumably leading to greater direct anthropogenic impacts (e.g., nutrient additions) relative to the more isolated reefs of Lee Stocking Island. Despite this, corals of the Exuma Cays recently experienced greater extremes of high temperature and bleached more often (e.g., in 2005 [19], [20]) than colonies from the Upper Florida Keys. Greater bleaching-induced stress and the subsequent years of recovery may override latitudinal effects, resulting in reduced tissue biomass and *Symbiodinium* density in corals from the Bahamas relative to Floridian conspecifics (see discussion of bleaching below).

### Tissue biomass and Symbiodinium densities vary with depth

Deeper corals often have lower tissue biomass and *Symbiodinium* density relative to individuals of the same species in shallow water [18], [31]. Previous studies attributed this pattern to photoacclimation by *Symbiodinium* populations to attenuating light [18], [29] and this explanation is consistent with our data for *M. faveolata*. In contrast, *M. annularis* experienced depth-related differences in tissue biomass, but not *Symbiodinium* density. Furthermore, *A. cervicornis* showed either no difference in tissue biomass or *Symbiodinium* density among depths (in Florida), or an inverse relationship between these two parameters (in the Bahamas). These contrasting results suggest that additional factors contribute to bathymetric tissue biomass and symbiont density patterns. Compared to shallow habitats, deeper reefs are characterized by reduced light intensity, lower average temperature, and marked differences in currents and advective flow from deeper waters. Such physical patterns could drive differences in quantity and quality of zooplankton, detritus, and DOM that may also influence coral biomass. For example, certain corals increase heterotrophic intake with decreasing light intensity and increasing depth [24], [25]. Thus, biomass differences noted here are likely due, in part, to resource acquisition via autotrophy and heterotrophy, as many corals display a greater reliance on heterotrophically derived carbon and nitrogen in deeper waters [32], [33]. While differences in endosymbiont density, carbon translocation, and *Symbiodinium* genotype (mentioned below) are probable causes of the depth-related patterns in tissue biomass, more detailed comparative analyses are necessary to further understand corals' carbon and nutrient budgets.

Many corals harbor genetically distinct *Symbiodinium* sp. and some of the species studied here have polymorphic symbioses with at least three different *Symbiodinium* genotypes. For example, members of the *M. annularis* species complex harbor internal transcribed spacer region 2 (ITS2) “types” A3 (Mexico), B1 (all three regions), B10 (Florida), B17 (Mexico), C3 (Florida), C7 (Mexico), C12 (Bahamas), and/or D1a (all three regions) and these “types” exhibit zonation by depth and colony position [13], [20], [28]. In shallow, high-light environments, clade A and B “types” dominate the tops of *Montastraea* spp. colonies; as depth increases, clade C “types” increase in abundance [28], [34]. Additionally, considerable genetic diversity exists within a given *Symbiodinium* ITS2 “type” [35]. Even when identical *Symbiodinium* ITS2 genotypes are found in shallow and deep environments, additional genetic, and possibly physiological, differences likely exist [20], [36]. Consequently, differences in endosymbiont populations may further contribute to the depth-associated differences in host tissue biomass observed here.

### Seasonality and bleaching in coral tissue biomass and Symbiodinium density

Previous studies demonstrated that symbiont density [17] and tissue biomass fluctuate seasonally, with intra-annual maximums in the winter/spring and minima in the summer/fall [18]. This pattern was further confirmed for the majority of coral species examined. In addition, some summers are characterized by excessive and/or prolonged periods of heating (many of which correspond to strong ENSO cycles) and result in greater declines in tissue biomass, symbiont populations, and photosynthetic capacity, leading to severe coral bleaching [37], [38]. Results also indicate that certain corals, such as *A. cervicornis*, *A. palmata, M. franksi*, and *P. astreoides*, experience minimal seasonal change in biomass and/or symbiont density.

Seasonal changes in temperature and solar insulation play an important role in modulating coral metabolic rate [18]. High temperatures increase metabolic rate, which influences the depletion of lipid reserves, animal biomass, and skeletal growth [39]. Importantly, most Caribbean coral species reproduce by single annual broadcast spawning events in the late summer (i.e., *Montastraea, Acropora,* and *S. siderea*), while others brood and release mature larvae several times over the year (i.e., *P. astreoides*) [40]. Energetic demands of reproduction would further reduce lipids and overall biomass, thus contributing to the typical fall minima in coral tissue. Likewise, the more frequent reproductive effort and thermally tolerant symbioses of *P. astreoides* [13] may dampen seasonal biomass fluctuation in this species.

We identified bleaching in the form of significant declines in *Symbiodinium* density in *M. annularis*, *M. faveolata*, *M. franksi*, *A. cervicornis*, and *S. siderea*. Whereas bleaching was seen in the Florida Keys and Mexico in 1998, we were unable to sample most of these locations during the peak of the event, with the exception of ADM reef. Similarly, although bleaching was noted in 2005, the lack of symbiont loss in *Montastraea* spp. from Mexico in our samples is most likely due to the late sampling time (October), after symbiont populations began recovery. Thus, while many corals appeared pale, this may have been due to a substantial reduction in photosynthetic pigments per algal cell as the remaining symbionts were photoacclimating to the higher post-bleaching light field *in hospite* [41]. Clearly, annual symbiont loss, and greater decline during bleaching, plays a significant role in dictating resource partitioning in corals. Bleaching reduces the proportion of carbon translocated from *Symbiodinium* spp. to the host coral, and as a result, corals must consume stored energy reserves [42]–[44]. Our data also show that such losses in biomass do not always immediately coincide with losses of *Symbiodinium*, suggesting a possible time lag [18]. This was particularly apparent in Mexico, where sampling occurred after the 2005 event. For *Montastraea* spp., symbiont densities were in the process of recovery during sampling, whereas tissue biomass levels were experiencing significant declines.

### Low biomass corals are susceptible to death following stress

Seasonal monitoring revealed a correlation between low coral tissue biomass and colony mortality among individuals of *M. annularis*, *M. franksi*, and possibly *A. cervicornis*. This result is supported by Loya et al. [6], who documented higher survivorship among thick-tissue Pacific scleractinians, relative to thinner-tissue species, during the 1998 bleaching event. Similarly, Hoegh-Guldberg [1] proposed that thicker-tissue corals would be more resistant to bleaching due to increased photo-protective shading. These findings suggest the following hypotheses: 1) tissue biomass is indicative of overall coral colony health, 2) tissue biomass is predictive of colony survival probability following periods of stress, such as high-temperature induced bleaching, and 3) each species of coral has a biomass minimum, below which colonies are more susceptible to disease and starvation.

Mortality among *Montastraea* spp. colonies monitored at deep reef sites correlated with individuals maintaining a low average biomass of approx. 4.6 mg×cm^−2^ for an extended period and then being subjected to thermal stress. In contrast, colonies of *Montastraea* spp. with higher biomasses survived (Fig. 7). Similar observations were made for *A. cervicornis*, which exhibited low tissue biomass throughout the year at all locations sampled (Fig. 5d). At three reefs, *A. cervicornis* colonies died following either the 1997–1998 El Niño or putative disease incidents. These colonies experienced prolonged periods of low tissue biomass prior to their death (Fig. 8). While surviving *A. cervicornis* colonies exhibited slightly higher biomass values, all colonies experienced major reductions in the area of living coral tissue on their skeletons and many colonies died over the course of our sampling. Because all *A. cervicornis* colonies died or shrank substantially in tissue area (i.e., were dying), it was not possible to precisely estimate a minimum tissue threshold for this species. Based on comparisons to *A. palmata*, we estimated the minimum tissue threshold for *A. cervicornis* to be between 3.3 and 5.3 mg tissue×cm^−2^. However, comparisons between *Acropora* spp. should be interpreted cautiously, as the biomass differences may result primarily from intrinsic properties of each species as opposed to dissimilarity in colony health.

Based on the observations and correlations outlined above, we propose that corals that are unable to replenish their nutrient and energy reserves to a healthy level become metabolically fragile, and are thus increasingly susceptible to dying during episodes of stress when metabolic reserves are most needed. If this hypothesis is correct, tissue biomass represents a potentially useful indicator of the health of different corals. Field survey studies of mass bleaching events have noted differential mortality among coral colonies, species, and reefs [6], [9], [10]. This variability has been partially attributed to genetic and physiological differences among corals or their endosymbionts [11]–[13], however, such explanations do not account for all available data. Evidence presented here suggests that differences in colony biomass also contribute to the mortality patterns in reef-building corals and may in some cases underlie differential survival following bleaching.

## Materials and Methods

### Study site and collection of corals

This study targeted seven species of corals, including *M. annularis*, *M. faveolata*, *M. franksi*, *A. cervicornis*, *A. palmata*, *P. astreoides*, and *S. siderea* for seasonal monitoring. Each coral species was sampled based on its local availability from up to three reefs in the Exuma Cays, Bahamas, five reefs in the Upper Florida Keys, United States (U.S.), and one reef in Puerto Morelos, Mexico. Site coordinates, depth, and coral species collected at each site are provided in Table 1 and a map of the collection localities is shown in Fig. 1. Each reef was sampled seasonally, with sample periods designated here as winter (January–March), spring (April–June), summer (July–September), and fall (October–December). Occasionally, circumstances (e.g., storm activity) prohibited the collection of samples during a particular time point, and as a result, collection frequency ranged between two to four samplings per site per year. Collection durations at each site are listed in Table 1.

At each site, six representative colonies of each species were tagged at the beginning of the study to ensure that subsequent collections were from the same coral colony. Due to the difficulty in permanently tagging the branches of *A. cervicornis*, this coral was sampled from up to six distinct colonies from the same population at every collection period, but not necessarily the same colonies each time. (Note: sample sizes of *A. cervicornis* at deeper sites were reduced [n = 2–3] due to its rarity at depth.) At each sampling interval, approximately 10-cm^2^ fragments were removed from each colony using a hammer and chisel or punch core. All colony fragments were taken from non-shaded areas at or near the tops of each colony in order to minimize potential light micro-environmental variability effects across the colony surface. Fragments were placed in pre-labeled plastic bags filled with seawater and transported in an insulated cooler to the laboratory where they were processed immediately.

### Determination of coral tissue biomass and Symbiodinium density

Each coral fragment was split into two pieces. The first sub-fragment was processed for coral tissue biomass following a modified protocol based on Johannes and Wiebe [45]. Tissue was removed from the skeleton with a recirculating Waterpik™ using distilled water and then frozen at −20°C. The frozen slurry was then lyophyilized and subsequently baked in a muffle furnace (Fisher Scientific) for at least 4 h at 500°C. Ash-free dry weight was calculated as the difference between dry weight and ash weight following the protocol of Fitt et al. [18]. The second coral sub-fragment was processed to determine *Symbiodinium* densities. This fragment was ‘waterpiked’ with 0.45 µm-filtered seawater. Algal densities were calculated from replicate (*n* = 6–10) hemocytometer counts of homogenized tissue. Fragment surface area was determined by the aluminum foil method [46] and tissue biomass and symbiont density were normalized to the surface area of the coral sample. The resulting raw data are publically available at the database DRYAD (http://datadryad.org; doi:10.5061/dryad.gm005fg8).

### Statistical analyses

We used Pearson correlations to determine the degree to which tissue biomass and *Symbiodinium* density were correlated in each of the seven coral species across locations, depths, and collection time points. Assumptions of normality and homogeneity of variance were tested and, because all data lacked normality, each dataset was square root transformed to satisfy normality assumptions. Unless otherwise noted, all subsequent statistical tests were performed on the transformed datasets.

For the following analyses, coral tissue biomass and *Symbiodinium* density were examined independently of one another using the analytical software JMP version 8 (SAS Institute Inc., Cary, NC, U.S.) and Systat version 13 (Cranes Software International, Bangalore, India). The effects of region, depth, and their interaction on tissue biomass or *Symbiodinium* density were tested using two-factor analyses of variance (ANOVA) and Tukey's honestly-significant-difference tests (*M. annularis*, *M. faveolata*, *A. cervicornis*, and *P. astreoides*). When total population size was less than 3 across all sites, T-tests were used instead (*M. franksi, A. palmata,* and *S. siderea*). We used separate two-factor ANOVAs to test for effects on coral tissue biomass and *Symbiodinium* density instead of a single MANOVA because tissue biomass and symbiont density were often correlated (see results), thus violating assumptions of colinearity of variances in dependent variables. For all seven coral species, the effects of location, season, and their interaction on tissue biomass or *Symbiodinium* density were tested using two-factor ANOVAs. Changes in tissue biomass and *Symbiodinium* density coincident with bleaching events were examined by comparing parameter values during the bleaching event to the mean summer biomass or symbiont density value for that reef and species, excluding bleaching year data, using two-sample protected T-tests. The mean summer value was used for these comparisons because this is the period in which tissue biomass and *Symbiodinium* density are typically the lowest each year [18].

To test the hypothesis that low tissue biomass renders coral colonies susceptible to mortality, optimal mortality thresholds were determined within a differential range separating colony outcomes (survival versus mortality). The method (see Text S1) identified the single threshold value in the range between greatest “dying” value and lowest “surviving” value that had the lowest probability of incorrectly classifying a coral as surviving or dying based on its actual measured tissue biomass and known fate (given that the data were normally distributed). Thresholds were tested in increments of 0.1 across the differential range; threshold values were considered to be acceptable if their probability of misclassification was less than 0.05. Thus, mortality threshold analysis resulted in a single optimum value and range of acceptable values for each differential range tested.

## Supporting Information

Table S1 **Two-factor ANOVA with interaction terms summary based on the biomass and** ***Symbiodinium*** **densities of seven corals.**
(DOCX)

Text S1 **Protocol and programs for mortality threshold analysis, designed and calculated with MATLAB R2007b.**
(DOCX)
