# Supplementary material for: A Connection between Colony Biomass and Death in Caribbean Reef-Building Corals
Source: PLoS One. 2011 Dec 22;6(12):e29535. doi: 10.1371/journal.pone.0029535 (PMC3245285; doi:10.1371/journal.pone.0029535)
Supplement: Table S1 — Two-factor ANOVA with interaction terms summary based on the biomass and Symbiodinium densities of seven corals. (DOCX) [file pone.0029535.s001.docx]

**Table S1**: Two-factor ANOVA with interaction terms summary based on the tissue biomass and *Symbiodinium* densities of seven scleractinian species.

| **Scleractinian species** | **Parameter** | **Source of Variation** | **Type III SS** | **df** | **Mean Squares** | **F-ratio** | ***p* value** |
| --- | --- | --- | --- | --- | --- | --- | --- |
| *M. annularis* | Tissue biomass | Reef | 56.582 | 7 | 8.083 | 40.721 | < 0.0001 |
|  |  | Season | 8.969 | 3 | 2.990 | 15.061 | < 0.0001 |
|  |  | Reef*Season | 10.157 | 21 | 0.484 | 2.437 | < 0.0001 |
|  |  | Error | 205.650 | 1,036 | 0.199 |  |  |
|  | Symbiont density | Reef | 23.006 | 7 | 3.287 | 29.700 | < 0.0001 |
|  |  | Season | 5.731 | 3 | 1.910 | 17.262 | < 0.0001 |
|  |  | Reef*Season | 7.592 | 21 | 0.362 | 3.267 | < 0.0001 |
|  |  | Error | 117.960 | 1,066 | 0.111 |  |  |
| *M. faveolata* | Tissue biomass | Reef | 72.738 | 7 | 10.391 | 45.133 | < 0.0001 |
|  |  | Season | 14.937 | 3 | 4.979 | 21.625 | < 0.0001 |
|  |  | Reef*Season | 8.298 | 21 | 0.395 | 1.716 | 0.023 |
|  |  | Error | 240.596 | 1,045 | 0.230 |  |  |
|  | Symbiont density | Reef | 66.380 | 7 | 9.483 | 58.129 | < 0.0001 |
|  |  | Season | 17.229 | 3 | 5.743 | 35.204 | < 0.0001 |
|  |  | Reef*Season | 10.858 | 21 | 0.517 | 3.170 | < 0.0001 |
|  |  | Error | 180.914 | 1,109 | 0.163 |  |  |
| *M. franksi* | Tissue biomass | Reef | 5.480 | 3 | 1.827 | 11.225 | < 0.0001 |
|  |  | Season | 0.427 | 3 | 0.142 | 0.874 | 0.455 |
|  |  | Reef*Season | 1.552 | 9 | 0.172 | 1.059 | 0.392 |
|  |  | Error | 69.005 | 424 | 0.163 |  |  |
|  | Symbiont density | Reef | 0.628 | 3 | 0.209 | 2.105 | 0.099 |
|  |  | Season | 1.239 | 3 | 0.413 | 4.154 | 0.006 |
|  |  | Reef*Season | 2.647 | 9 | 0.294 | 2.959 | 0.002 |
|  |  | Error | 43.549 | 438 | 0.099 |  |  |
| *A.cervicornis* | Tissue biomass | Reef | 2.342 | 6 | 0.390 | 4.801 | <0.001 |
|  |  | Season | 0.232 | 3 | 0.077 | 0.952 | 0.415 |
|  |  | Reef*Season | 3.100 | 17 | 0.182 | 2.243 | 0.003 |
|  |  | Error | 34.627 | 426 | 0.081 |  |  |
|  | Symbiont density | Reef | 18.643 | 6 | 3.107 | 42.938 | < 0.001 |
|  |  | Season | 0.672 | 3 | 0.224 | 3.094 | 0.027 |
|  |  | Reef*Season | 1.468 | 17 | 0.086 | 1.193 | 0.266 |
|  |  | Error | 31.334 | 433 | 0.072 |  |  |
| *A.palmata* | Tissue biomass | Reef | 1.083 | 1 | 1.083 | 6.283 | 0.013 |
|  |  | Season | 2.918 | 3 | 0.973 | 5.640 | 0.001 |
|  |  | Reef*Season | 0.430 | 3 | 0.143 | 0.831 | 0.477 |
|  |  | Error | 64.498 | 374 | 0.172 |  |  |
|  | Symbiont density | Reef | 17.233 | 1 | 17.233 | 126.837 | < 0.0001 |
|  |  | Season | 0.646 | 3 | 0.215 | 1.585 | 0.193 |
|  |  | Reef*Season | 0.518 | 3 | 0.173 | 1.270 | 0.284 |
|  |  | Error | 53.530 | 394 | 0.136 |  |  |
| *P. astreoides* | Tissue biomass | Reef | 9.760 | 2 | 4.880 | 24.646 | < 0.0001 |
|  |  | Season | 1.278 | 3 | 0.426 | 2.151 | 0.096 |
|  |  | Reef*Season | 7.242 | 6 | 1.207 | 6.096 | < 0.0001 |
|  |  | Error | 34.649 | 175 | 0.198 |  |  |
|  | Symbiont density | Reef | 6.645 | 2 | 3.322 | 36.117 | < 0.0001 |
|  |  | Season | 2.509 | 3 | 0.836 | 9.091 | < 0.0001 |
|  |  | Reef*Season | 5.146 | 6 | 0.858 | 9.324 | < 0.0001 |
|  |  | Error | 16.466 | 179 | 0.092 |  |  |
| *S. siderea* | Tissue biomass | Reef | 8.056 | 2 | 4.028 | 21.843 | < 0.0001 |
|  |  | Season | 2.090 | 3 | 0.697 | 3.777 | 0.011 |
|  |  | Reef*Season | 7.083 | 6 | 1.180 | 6.401 | < 0.0001 |
|  |  | Error | 41.307 | 224 | 0.184 |  |  |
|  | Symbiont density | Reef | 1.584 | 2 | 0.792 | 6.423 | 0.002 |
|  |  | Season | 7.524 | 3 | 2.508 | 20.343 | < 0.0001 |
|  |  | Reef*Season | 3.919 | 6 | 0.653 | 5.298 | < 0.0001 |
|  |  | Error | 28.848 | 234 | 0.123 |  |  |
