# Supplementary material for: A Connection between Colony Biomass and Death in Caribbean Reef-Building Corals
Source: PLoS One. 2011 Dec 22;6(12):e29535. doi: 10.1371/journal.pone.0029535 (PMC3245285; doi:10.1371/journal.pone.0029535)
Supplement: Text S1 — Protocol and programs for mortality threshold analysis, designed and calculated with MATLAB R2007b. (DOCX) [file pone.0029535.s002.docx]

**Text S1**: Protocol and programs for mortality threshold analysis, designed and calculated with MATLAB R2007b.

To determine the optimum threshold for separating two sets of values (e.g., survival versus mortality), the following algorithm was employed, implemented with a custom MATLAB script:

1. Potential thresholds were bounded by the greatest value in the “lower” data set and the least value in the “higher” data set.
2. Potential thresholds were considered in certain increments within this interval.
3. For a particular potential threshold value *T*, the probability *P_i_*(*T*) that the threshold was actually part of a normal distribution for each measured value *i* (defined by its mean *x_i_* and standard deviation *σ_i_*) was determined, by integrating from the threshold to the appropriate infinity (defined as integration away from the center of the distribution, negative infinity for the “higher” set and positive infinity for the “lower” set). The result of this integration is defined by the error function complement (*erfc*), and is given by

$$P_{i}(T)=\frac{1}{2}erfc\left(\frac{1}{2}\frac{\left|x_{i}-T\right|}{\sigma_{i}}\right)$$

This number may be regarded as the probability that subsequent measurements from the same population will lie on the different side of the threshold as the reported mean measurement; in this case, the selected threshold would misclassify the new sample.

1. For each potential threshold *T*, the joint probability *P’*(*T*) of correctly classifying all colonies (assuming coral colony measurements to be independent of each other) was recorded:

$$P'(T)=\prod_{i} \left(1-P_{i}(T)\right)$$

and the complementary probability of incorrectly classifying at least one coral colony *P*(*T*) is

$$P\left( T \right)=1-P'(T)$$

1. Plots of this probability of threshold error as a function of potential threshold were generated. The threshold value with the minimum error was defined as the optimum threshold. A band of “acceptable” thresholds was further defined by using a probability *α* of 0.05; any threshold within this band may be taken as a value with 5% or less chance of incorrectly classifying any sample.

Specific results: (A) The value of 4.6 mg tissue × cm^-2^ was found to be the optimal threshold for *Montastraea* spp. (B) Threshold analysis was also conducted for *Acropora* spp., and a range was determined between the values of 2.9 and 5.6 mg tissue × cm^-2^. This range covered the maximum observed among dying *A. cervicornis* (at NP and PB) and the minimum of surviving *A. palmata* (at PB) respectively. For *Acropora* spp., values ranging between 3.3 and 5.3 mg tissue × cm^-2^ represented acceptable thresholds for mortality; the optimal threshold was 4.0 Optimum threshold identified is indicated by the arrow below; dotted line represents the probability *α* cutoff of 0.05.

1. Potential thresholds taken from 4.3 to 5.5 in increments of 0.1; no acceptable thresholds (minimum error probability 0.0856 at the threshold 4.7).

%%threshfind.m--find optimum threshold to separate data

means=[5.933; 4.251; 6.046; 5.928; 7.336; 5.295; 7.607; 8.001; 6.501; 4.188; 5.542; 5.711];

SEs=[0.133; 0.137; 0.377; 0.18; 0.174; 0.261; 0.268; 0.269; 0.188; 0.164; 0.229; 0.139];

counter=1;

for threshold=4.3:.1:5.5;

for loop=1:length(means)

p(loop)=0.5*erfc(0.5*abs(means(loop)-threshold)/SEs(loop));

end

totp(counter)=1-prod(1-p);

counter=counter+1;

end

plot(4.3:.1:5.5, totp)

xlabel('threshold T')

ylabel('misclassification probability P(T)')

%minimum P(T): 0.0856 (T=4.7)

%0.05 cutoff: no thresholds below this level

1. Consider all *A. cervicornis* colonies dying: potential thresholds taken from 2.9 to 5.6 in increments of 0.1; optimal threshold 4.5; acceptable thresholds 3.3-5.3.

%%threshfind2.m--find optimum threshold to separate data

means=[2.853; 2.857; 2.262; 2.693; 2.385; 1.936; 2.622; 5.679; 6.313];

SEs=[0.097; 0.158; 0.097; 0.089; 0.091; 0.157; 0.225; 0.138; 0.167];

counter=1;

for threshold=2.9:.1:5.6;

for loop=1:length(means)

p(loop)=0.5*erfc(0.5*abs(means(loop)-threshold)/SEs(loop));

end

totp(counter)=1-prod(1-p);

counter=counter+1;

end

close all

semilogy(2.9:.1:5.6, totp)

xlabel('threshold T')

ylabel('misclassification probability P(T)')

%minimum P(T): 2.561E-9 (T=4.5)

%0.05 cutoff: 3.3-5.3
